# Supplementary material for: The use of feasibility studies for stepped-wedge cluster randomised trials: protocol for a review of impact and scope
Source: BMJ Open. 2017 Aug 1;7(7):e017290. doi: 10.1136/bmjopen-2017-017290 (PMC5642661; doi:10.1136/bmjopen-2017-017290)
Supplement: Author's manuscript [file bmjopen-2017-017290.draft_revisions.pdf]

BMJ Open is committed to open peer review. As part of this commitment we make the peer review history of every article we publish publicly available.

When an article is published we post the peer reviewers' comments and the authors' responses online. We also post the versions of the paper that were used during peer review. These are the versions that the peer review comments apply to.

The versions of the paper that follow are the versions that were submitted during the peer review process. They are not the versions of record or the final published versions. They should not be cited or distributed as the published version of this manuscript.

BMJ Open is an open access journal and the full, final, typeset and author-corrected version of record of the manuscript is available on our site with no access controls, subscription charges or pay-per-view fees (http://bmjopen.bmj.com).

If you have any questions on BMJ Open's open peer review process please email editorial.bmjopen@bmj.com

BMJ Open

The use of feasibility studies for stepped-wedge cluster randomised trials: a protocol for a review of impact and scope.

Journal:	*BMJ Open*
Manuscript ID	bmjopen-2017-017290
Article Type:	Protocol
Date Submitted by the Author:	12-Apr-2017
Complete List of Authors:	Kristunas, Caroline; University of Leicester, Department of Health Sciences<br>Hemming, Karla; University of Birmingham, Institute of Applied Health Research<br>Eborall, Helen; University of Leicester, Department of Health Sciences<br>Gray, Laura; University of Leicester, Department of Health Sciences
**Primary Subject Heading**:	Research methods
Secondary Subject Heading:	Public health
Keywords:	STATISTICS & RESEARCH METHODS, Stepped-wedge trial, Pilot and feasibility studies

SCHOLARONE™ Manuscripts

For peer review only

The use of feasibility studies for stepped-wedge cluster randomised trials: a protocol for a review of impact and scope

Caroline A Kristunas[1], Karla Hemming[2], Helen C Eborall[1], Laura J Gray[1]*

Author Affiliations:

[1]Department of Health Sciences, University of Leicester, Leicester, UK, LE1 7Rh.

[2]Institute of Applied Health Research, University of Birmingham, Birmingham, UK, B15 2TT.

**Corresponding author:* Laura J Gray; lg48@le.ac.uk, Department of Health Sciences, College of Medicine, Biological Sciences and Psychology, University of Leicester, Centre for Medicine, University Road, Leicester, LE1 7RH

For peer review only

ABSTRACT

Introduction: The stepped-wedge cluster randomised trial (SW-CRT) is a complex design, for which many decisions about key design parameters must be made during the planning. These include the number of steps and the duration of time needed to embed the intervention. Feasibility studies are likely to be useful for informing these decisions and increasing the likelihood of the main trial's success. However, how often feasibility studies are conducted for SW-CRTs is currently unknown. This review aims to establish how often feasibility studies are being conducted for SW-CRTs and determine which feasibility issues are commonly investigated.

Methods and analysis: Fully published feasibility studies for SW-CRTs will be identified, according to pre-defined inclusion criteria, from searches conducted in Ovid MEDLINE, Scopus, EMBASE, and PsycINFO. In order to also identify and gain information on unpublished feasibility studies the following will be contacted: authors of published SW-CRTs (identified from the most recent systematic reviews); contacts for registered SW-CRTs (identified from clinical trials registries); lead statisticians of UK registered Clinical Trials Units; and researchers known to work in the area of SW-CRTs.

Data extraction will be conducted independently by two reviewers. For the fully published feasibility studies, data will be extracted on the general characteristics, the rationale for the study, the process for determining progression to a main trial, how the study informed the main trial and whether the main trial went ahead. The researchers involved in the unpublished feasibility studies will be contacted in order to elicit the same information.

A narrative synthesis will be conducted and provided alongside a descriptive analysis of the study characteristics.

Ethics and dissemination: This review does not require ethical approval, as no individual patient data will be used. The results of this review will be published in an open access pee-reviewed journal.

Strengths and limitations:

- This review will be the first to provide an insight into how feasibility studies are being used to inform SW-CRTs.
- This review will identify both published and unpublished feasibility studies for SW-CRTs.
- In order to ensure a robust review and minimise potential bias, the search strategy and inclusion and exclusion criteria have been pre-specified.
- Although steps have been taken to minimise potential sources of bias, selection bias may be introduced through the exclusion of non-English language studies.

For peer review only

INTRODUCTION

The stepped-wedge cluster randomised trial

The stepped-wedge cluster randomised trial (SW-CRT) is seeing an unprecedented increase in its use.[1-4] For this design, clusters are randomised to start the intervention at different time points. All of the clusters start the trial not receiving the intervention, and then switch to the intervention at their allocated start time, until by the end of the trial all of the clusters are receiving the intervention [Figure 1].[5]

There are several appeals of the SW-CRT. One of the main appeals being that it allows a staggered introduction of the intervention, allowing clusters to act as their own control and for all clusters to eventually receive the intervention.[2, 6] In addition, there are occasions when the SW-CRT is more efficient than a parallel CRT, requiring a smaller sample size and fewer clusters.[7, 8] The SW-CRT can thus allow the experimental assessment of the effectiveness of certain interventions that, due to practical, logistical or financial reasons, it may not be possible to assess using another design of trial.[5, 8, 9] An example of a study for which the SW-CRT was considered the best option is the SANDWICH trial.[10] Details of the reasons for this choice of design and the decision processes related to this choice are described in Figure 2.

Trial decision process in the SW-CRT

The staggered nature of the introduction of clusters to the intervention in a SW-CRT requires many decisions to be made during the design process, such as the number of start times and the length of time between start times.[8] For example there may be logistical constraints that mean it is only feasible to switch a certain number of clusters at once, or the intervention may take a while to embed in a cluster, in which case the length of time between start times might need to be sufficiently long to allow for this.[8] Alternatively, the trial might need to be completed within a fixed funding period, in which case the step length will need to be short enough to allow all clusters to switch within this period. These decisions are in addition to those that must be made for a standard CRT, and are unlikely to be able to be informed by previous studies. It is known that, although advantageous is some respects, the staggered introduction of the intervention may increase the complexity of the trial in both practical and statistical terms.[6, 8] Some of the issues associated with a staggered implementation are being investigated as part of a feasibility study for the SANDWICH trial, details of which are given in Figure 2.

What feasibility and pilot studies may offer

The terms pilot and feasibility study are often used interchangeably to describe a small scale study, that is conducted prior to the main trial, and which aims to guide the planning or design of the trial, or to confirm its feasibility.[11-14] When designing a trial, decisions may be informed by previous trials, systematic reviews, routine data etc., but there may be aspects of the trial that require additional information which might only be obtained through the use of a feasibility or pilot study.[13] Pilot or feasibility studies are becoming the norm prior to a definitive individually randomised trial, and test trial factors such as recruitment, retention and acceptability of the intervention.[15, 16]

For peer review only

Cluster randomised trials (CRTs) are typically larger and more expensive, and due to their additional complexity, tend to need more assumptions and decisions to be made at the design stage than for an individually randomised trial.[13, 17] Each aspect of the design that is assessed at an individual-level, now needs to also be assessed at the cluster-level. These may include recruitment and retention rates or the acceptability of the intervention to both the individuals and the clusters. Specifically for CRTs, an estimate of the extent of variation between clusters (intracluster correlation coefficient (ICC)) is needed in order to calculate the required sample size. However, Eldridge et al. [17] have shown that feasibility studies to investigate ICCs for CRTs will often have insufficient accuracy, unless the feasibility study is almost as large as the main trial.

Feasibility studies may be of even greater potential benefit for SW-CRTs than they are for standard CRTs, as numerous additional decisions must be made. These include determining the number of clusters it is feasible to switch to the intervention at each step and the length of time between each step needed to embed the intervention, ensuring that the full effect of the intervention is realised and enough participants are recruited for each stage of the trial.[9]

Previous systematic reviews have investigated the reasons why a pilot or feasibility study might be undertaken for a randomised controlled trial (RCT),[11, 15, 18] but these reviews are unlikely to have captured many pilot or feasibility studies for SW-CRTs. In addition, no published systematic reviews of SW-CRTs have included feasibility studies, so it is not currently known to what extent feasibility studies are being conducted for SW-CRTs, nor what issues these studies are being designed to investigate.

<u>What our review will achieve</u>

We aim to identify what feasibility work is being conducted for SW-CRTs. This will be achieved through a systematic review of the published literature, which will identify published feasibility studies for SW-CRTs. However, as feasibility studies often go unpublished, we will extend our review to identify unpublished feasibility studies. This will be achieved by contacting the authors of published and registered SW-CRTs, lead statisticians of UK registered Clinical Trials Units (CTUs) and researchers who are known by the authors to work in the area of SW-CRTs.

OBJECTIVES

The overarching aims of this review are to determine how often feasibility studies are conducted for SW-CRTs and how these studies are being used to inform the subsequent SW-CRTs. Specifically, our objectives are to:

1. Systematically identify published feasibility studies for SW-CRTs;
2. Identify unpublished feasibility work that has been conducted or is planned to take place prior to a SW-CRT;
3. Extract information on the design characteristics and rationale for these feasibility studies, as well as how these studies inform the main trial and how the decision is made as to whether to progress to the main trial.

For peer review only

METHODS

A two-pronged approach will be implemented to identify feasibility studies for SW-CRTs. Published feasibility studies will be identified through a systematic review of the literature, whereas unpublished feasibility studies will be identified by contacting researchers who may have been involved in, or have knowledge of, feasibility studies for SW-CRTs that have been conducted but which have not yet been published.

Search strategy

Identification of published feasibility studies for SW-CRTs

We will identify eligible feasibility studies for SW-CRTs, published in English, via electronic searches of the online published databases Ovid MEDLINE (1946-present), Scopus (1966-present), EMBASE (1947-present) and PsycINFO (1967-present). The initial search strategy will include a combination of MeSH terms and keywords specific to each bibliographic database. An example search strategy is outlined in Table 1 and is based on previously published search strategies.[1-3, 11, 15]

The titles and abstracts for the identified studies will be screened for eligibility by two reviewers independently in a random order, with full-text articles being obtained for potentially eligible studies and the same duplicate method of assessment used. Any studies which are not found to be eligible will be excluded and the reason for exclusion noted. Any differences of opinion will be resolved by a third reviewer if required. Authors will be contacted if any further information is required and we will aim to access any published or unpublished protocols for each of the identified feasibility studies.

Table 1: Example search strategy for Ovid MEDLINE

1. “pilot*”.mp
2. “feasibil*”.mp
3. 1 OR 2
4. “step* wedge*”.mp
5. “step*wedge*”.mp
6. “delay* intervention”.mp
7. “experimental* staged introduction”.mp
8. (“one* direction* crossover design” OR “one* direction* cross* over design”).mp
9. (“incremental* recruitment” OR “incremental* introduction” OR “incremental* implementation” OR “incremental* allocation”).mp
10. (“phased* recruitment” OR “phased* introduction” OR “phased* implementation” OR “phased* allocation”).mp
11. (“staggered* recruitment” OR “staggered* introduction” OR “staggered* implementation” OR “staggered*allocation”).mp
12. (“stepwise* recruitment” OR “stepwise* introduction” OR “stepwise* implementation” OR “stepwise*allocation”).mp

For peer review only

13. (“step*wise* recruitment” OR “step*wise* introduction” OR “step*wise* implementation” OR “step*wise*allocation”).mp
14. (“delayed* recruitment” OR “delayed* introduction” OR “delayed* implementation” OR “delayed*allocation”).mp
15. or/4-14
16. 3 AND 15
17. limit 16 to English language

Reference lists of the identified studies will also be checked for potentially eligible studies. Any additional published feasibility studies for SW-CRTs which are known to the authors, but which are not identified during the database searches, will also be included and assessed for eligibility. Records will be managed using Refworks reference management software.

<u>Identification of unpublished feasibility studies for SW-CRTs</u>

Feasibility studies for SW-CRTs might not always be published. In an attempt to identify the anticipated majority of these studies, our systematic review will be extended to allow inclusion of unpublished feasibility studies. This will be achieved in the following ways:

- Unpublished feasibility work for published SW-CRTs will be identified from the SW-CRTs reported in the most recent systematic reviews. The trial reports and references will be examined for evidence of feasibility work. The authors of these trials will then be contacted to identify whether any unreported feasibility work was conducted.
- Feasibility work that has been conducted for SW-CRTs that are yet to be published will be sought by contacting the lead statisticians of CTUs, researchers working in the area of stepped-wedge trials, as well as through searches of clinical trials registries.

Inclusion and exclusion criteria

For the systematic review of published feasibility studies, eligible studies will be full reports or protocols of feasibility studies for SW-CRTs. For the unpublished feasibility studies no publications will be required in order to qualify for inclusion.

The definition of, and distinction between feasibility and pilot studies is not always clear, with the terms often being used interchangeably [11, 12] and conflicting definitions existing for the distinction between the terms.[14, 19] For the purpose of this review (including both published and unpublished studies) a feasibility study will be defined as a study, with clearly defined aims and objectives, which intends to ascertain the feasibility of a planned SW-CRT, through the assessment of issues other than the effectiveness and refinement of the intervention, such as recruitment. Since pilot studies will also often investigate issues affecting the feasibility of the main trial, they will be considered to be a type of feasibility study for the purpose of this review, provided they satisfy the criteria of a feasibility study.

The feasibility study itself does not need to be of a stepped-wedge design, or even randomised, as the design will depend on the objectives of the study. However, the study should, through focussed objectives, make it clear how the findings of the feasibility study will inform the main study, which must be intended to be of a SW-CRT design. A SW-CRT will be defined as a randomised trial, which randomises clusters and which has two or more steps (time-points at which clusters change treatment group). Studies where the planned definitive trial is individually randomised, has a bi-directional cross-over design or is non-randomised, will be excluded. Neither the published nor the unpublished feasibility studies will be limited to healthcare settings.

The researchers involved in the unpublished feasibility studies that we identify will be contacted to gain the information required to determine eligibility.

Data extraction

Data for eligible published feasibility studies will be extracted by two reviewers independently and in random order using a data extraction form that will have been tested on a small number of studies, before being refined and finalised. Any differences will be resolved though discussion with a third reviewer. Extracted data will be managed in Microsoft Excel 2013.

For the eligible unpublished feasibility studies, data will be obtained through semi-structured interviews with the researchers involved in the studies. This will be conducted by a single reviewer. The same data will be collected for the unpublished feasibility studies as is extracted for the published feasibility studies.

<u>Data extraction of trial characteristics</u>

Data will be extracted on aspects of the design of the feasibility study, including the randomisation, blinding and overall design (parallel, stepped-wedge etc.). Data will also be extracted on how each feasibility study defines itself (either as a pilot or feasibility study), the size of the feasibility study and how the chosen sample size is justified.

<u>Data extraction of study rationale</u>

Data will be extracted on the rationale for conducting a feasibility study prior to the main trial. This will be achieved by extracting the specific aims of each study and categorising them into process, resource, management and scientific motivations.[20] These categories will be defined as:

- <u>Process-</u> assesses the feasibility of the steps that need to take place during the main study. This will include the estimation of recruitment, consent and retention rates, testing of the process and acceptability of the randomisation procedure, testing of the exclusion criteria, identification of barriers to recruitment, adherence etc.. Some of these will be specific to the SW-CRT, for example the recruitment rates per measurement period, which will determine the length of time between steps, whereas others will be more generic.
- <u>Resource-</u> assesses the time and budget problems that may occur during the main trial. This may include the time taken for data collection e.g. time taken to mail or fill

For peer review only - http://bmjopen.bmj.com/site/about/guidelines.xhtml

out forms/surveys and have them returned, or the time taken for data entry to be completed. The human resources available may also be a consideration, ensuring that the right people are available with the required expertise. Specific to SW-CRT, these aims might include the length of time required to rollout the intervention, or the time taken to acquire the necessary permissions from each of the clusters.

- Management- assesses the potential for human and data optimisation problems, such as the feasibility of international collaborations and across site coordination for multi-centre trials.
- Scientific motivations- assesses the scientific processes such as treatment safety and acceptability and estimation of the ICC (not recommended [17]), potential effectiveness and variance parameters.

In addition, the aims of each feasibility study will be sub-divided into aims that are specific to the SW-CRT and those which will be common with other trial designs.

Data extraction of progression to main trial

Data will be extracted on the process for deciding whether the study will progress to the main trial. This will include the type of analysis conducted, either hypothesis testing or descriptive, how much emphasis is put on the results of any hypothesis tests and the criteria used for determining success (main trial is considered feasible). Information on any hard or soft stopping rules that are in place for the feasibility studies will also be extracted.

Data extraction of main trial characteristics

Data will be extracted on the details of the main trials that follow each of the feasibility studies; whether it goes ahead, how the feasibility study has informed or made changes to the main trial and whether any of the participants who took part in the feasibility study also took part in the main trial.

Analysis of results

We will present a narrative synthesis of our findings, as well as a descriptive analysis of the study characteristics of each of the eligible feasibility studies that we identify for inclusion in the review.

Ethics and dissemination

As no individual patient data will be used, this review does not require ethical approval. We intend on presenting the findings of this review in an open-access peer-reviewed publication in an appropriate journal. We also intend on disseminating the findings through presentations at relevant conferences.

DISCUSSION

Although several reviews have been conducted in the area of stepped wedge trials,[1-4, 21, 22] none have investigated the use of feasibility studies for SW-CRTs. Due to their complexity, there are likely to be many issues that will affect the feasibility of SW-CRTs, some of which may be common across studies. However, it has yet to be reported how often feasibility work is being conducted prior to SW-CRTs nor what feasibility issues these

For peer review only

studies have been designed to inform. We aim to use our review to determine how often feasibility studies are conducted for SW-CRTs and what issues these studies have been designed to investigate.

By pre-specifying our search strategy, the inclusion and exclusion criteria and ensuring that two reviewers independently review all studies in a randomised order, we aim for our review to be robust, minimising potential sources of bias. Our review would be limited if relying solely on published feasibility studies for SW-CRTs, as many will not have been published, particularly if the main trial was found to be infeasible. However, by extending our systematic review to identify unpublished feasibility studies for SW-CRTs, we will be able to widen the reach of our review. We will contact authors of published SW-CRTs and researchers known to be working in the area of SW-CRTs to determine if they have knowledge of additional studies that we can include in our review. In addition, the term "stepped wedge" has not been universally used to describe a trial of this design and so we have included in our search strategy other terms used to describe this design, terms which have been used in previous reviews.[1-4, 22] There still remains the potential for selection bias in our review, as only trials using one of these terms will be included, which will exclude any SW-CRTs using alternative terminology. Finally, we may introduce selection bias through the exclusion of non-English language studies, although there is evidence that this has a minimal effect.[23]

CONCLUSION

This review is the first in a series of related projects investigating the feasibility of SW-CRTs. It will determine how often feasibility studies are being used to inform SW-CRTs and identify the feasibility issues that are being investigated and how these studies are informing the main SW-CRTs. This will provide an insight into how feasibility studies can benefit SW-CRTs. Future work will identify the feasibility issues being encountered in SW-CRTs and ultimately lead to the development of guidance on how feasibility studies for SW-CRTs can be conducted.

DECLARATIONS

Funding

This report is independent research supported by the National Institute for Health Research (NIHR Doctoral Research Fellowship, Miss Caroline Kristunas, DRF-2016-09-025. The authors would also like to acknowledge support from the NIHR Collaboration for Leadership in Applied Health Research and Care – East Midlands (NIHR CLAHRC – EM), NIHR CLAHRC – West Midlands, the Leicester Clinical Trials Unit and the NIHR Leicester-Loughborough Diet, Lifestyle and Physical Activity Biomedical Research Unit, which is a partnership between University Hospitals of Leicester NHS Trust, Loughborough University and the University of Leicester. The views expressed are those of the authors and not necessarily those of the NHS, the National Institute for Health Research or the Department of Health.

Contributors

For peer review only

321 CK conceptualised the review, the design of which was refined by incorporating comments from KH, HE and LG. CK drafted the manuscript and incorporated comments from KH, HE and LG for all subsequent drafts. All authors approved the final version and agree to be accountable for all aspects of the work.

325 **Data sharing**

326 No additional data are available.

327 **Competing interests**

328 We have read and understood BMJ policy on declaration of interests and declare that we have no competing interests.

330 **Copyright**

331 *The Corresponding Author has the right to grant on behalf of all authors and does grant on behalf of all authors, a worldwide licence to the Publishers and its licensees in perpetuity, in all forms, formats and media (whether known now or created in the future), to i) publish, reproduce, distribute, display and store the Contribution, ii) translate the Contribution into other languages, create adaptations, reprints, include within collections and create summaries, extracts and/or, abstracts of the Contribution, iii) create any other derivative work(s) based on the Contribution, iv) to exploit all subsidiary rights in the Contribution, v) the inclusion of electronic links from the Contribution to third party material where-ever it may be located; and, vi) licence any third party to do any or all of the above.*

340 **REFERENCES**

341 1 Brown CA, Lilford RJ. The stepped wedge trial design: a systematic review, *BMC medical research methodology* 2006;6:1.

343 2 Mdege ND, Man M, Taylor CA, et al. Systematic review of stepped wedge cluster randomized trials shows that design is particularly used to evaluate interventions during routine implementation, *J Clin Epidemiol* 2011;64:936-48.

346 3 Beard E, Lewis JJ, Copas A, et al. Stepped wedge randomised controlled trials: systematic review of studies published between 2010 and 2014, *Trials* 2015;16:1.

348 4 Grayling MJ, Wason JM, Mander AP. Stepped wedge cluster randomized controlled trial designs: a review of reporting quality and design features, *Trials* 2017;18:33.

350 5 Hussey MA, Hughes JP. Design and analysis of stepped wedge cluster randomized trials, *Contemporary clinical trials* 2007;28:182-91.

352 6 Prost A, Binik A, Abubakar I, et al. Logistic, ethical, and political dimensions of stepped wedge trials: critical review and case studies, *Trials* 2015;16:1.

354 7 Baio G, Copas A, Ambler G, et al. Sample size calculation for a stepped wedge trial, *Trials* 2015;16:1.

For peer review only

8 Hemming K, Haines TP, Chilton PJ, et al. The stepped wedge cluster randomised trial: rationale, design, analysis, and reporting, *BMJ* 2015;350:h391 doi:10.1136/bmj.h391.

9 Woertman W, de Hoop E, Moerbeek M, et al. Stepped wedge designs could reduce the required sample size in cluster randomized trials, *J Clin Epidemiol* 2013;66:752-8.

10 Anonymous . HTA - 15/104/01: Sedation and weaning in children: the SANDWICH trial. ;2017.

11 Arain M, Campbell MJ, Cooper CL, et al. What is a pilot or feasibility study? A review of current practice and editorial policy, *BMC Med Res Methodol* 2010;10:67.

12 Whitehead AL, Sully BGO, Campbell MJ. Pilot and feasibility studies: Is there a difference from each other and from a randomised controlled trial? *Contemporary Clinical Trials* 2014;38:130-3 doi:10.1016/j.cct.2014.04.001 [published Online First: May].

13 Eldridge S, Kerry S. A practical guide to cluster randomised trials in health services research: John Wiley & Sons 2012.

14 Eldridge SM, Chan CL, Campbell MJ, et al. CONSORT 2010 statement: extension to randomised pilot and feasibility trials, *Pilot and Feasibility Studies* 2016;2:64.

15 Lancaster GA, Dodd S, Williamson PR. Design and analysis of pilot studies: recommendations for good practice, *J Eval Clin Pract* 2004;10:307-12.

16 Anderson GL, Prentice RL. Individually randomized intervention trials for disease prevention and control, *Stat Methods Med Res* 1999;8:287-309.

17 Eldridge SM, Costelloe CE, Kahan BC, et al. How big should the pilot study for my cluster randomised trial be? *Statistical Methods in Medical Research* 2016;25:1039-56 doi:10.1177/0962280215588242 [published Online First: June 1,].

18 Shanyinde M, Pickering RM, Weatherall M. Questions asked and answered in pilot and feasibility randomized controlled trials, *BMC medical research methodology* 2011;11:117.

19 Eldridge SM, Lancaster GA, Campbell MJ, et al. Defining feasibility and pilot studies in preparation for randomised controlled trials: development of a conceptual framework, *PloS one* 2016;11:e0150205.

20 Thabane L, Ma J, Chu R, et al. A tutorial on pilot studies: the what, why and how, *BMC medical research methodology* 2010;10:1.

21 Barker D, McElduff P, D'Este C, et al. Stepped wedge cluster randomised trials: a review of the statistical methodology used and available, *BMC medical research methodology* 2016;16:1.

For peer review only

22 Martin J, Taljaard M, Girling A, et al. Systematic review finds major deficiencies in sample size methodology and reporting for stepped-wedge cluster randomised trials, *BMJ Open* 2016;6:010166.

23 Juni P, Holenstein F, Sterne J, et al. Direction and impact of language bias in meta-analyses of controlled trials: empirical study, *Int J Epidemiol* 2002;31:115-23.

For peer review only

Figure 1: Schematic of a stepped-wedge cluster randomised trial, with four steps and one cluster switching from control (white) to the intervention (light blue) at each time point. A recruitment period (light green) has been included prior to the first measurement period, as well as a bedding-in period (dark blue) during which the intervention will be embedded within the cluster. A cross-sectional sample is taken during each measurement period, at the time point indicated by the orange line, from which the outcome measure is obtained.

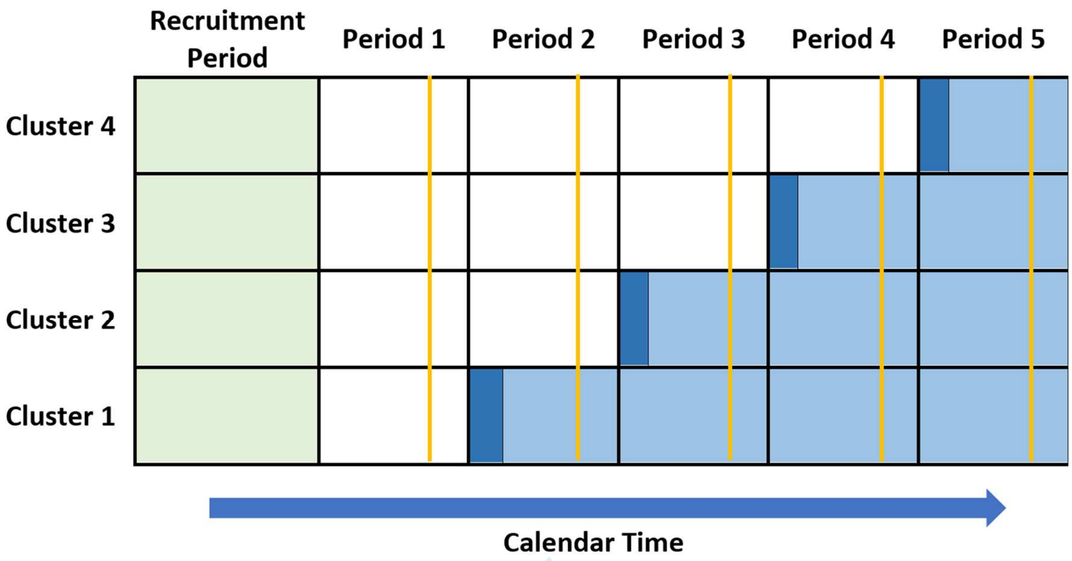

review only

Figure 2: A case study of the SANDWICH stepped-wedge cluster randomised trial.

Case study: the SANDWICH trial

The **S**edation **AND** **W**eaning **I**n **CH**ildren (SANDWICH) trial, will be a cluster-randomised stepped-wedge clinical and cost-effectiveness trial set in UK Paediatric Intensive Care Units (PICU). A protocol-based intervention, incorporating co-ordinated care with greater nursing involvement in managing sedation and weaning ventilation, will be assessed for its effectiveness at reducing the duration of invasive mechanical ventilation.

There are several reasons why a stepped-wedge cluster randomised design was chosen for the SANDWICH trial. Firstly, it was important that randomisation was undertaken at the cluster level, as the intervention will be delivered at the PICU (cluster) level and contamination would have occurred if individual-level randomisation had been used. Secondly, it would have been infeasible to conduct a conventional parallel cluster trial. There are a maximum of 20 PICUs in the UK, limiting the number of available clusters to such an extent that there would not have been a sufficient number to detect the important clinical effect at the desired 90% power.

It is anticipated that, due to the staggered rollout of the intervention, there will be particular issues that will need to be overcome. It is desired to conceal the allocation sequence, as advance knowledge of the allocation sequence can result in clusters withdrawing if they are allocated to switch to the intervention late in the trial. A feasibility study, taking the form of an internal pilot, will be used to determine how close the crossover date can be revealed to each cluster, to allow them sufficient time to prepare. This study will also aim to confirm the feasibility of data collection procedures and delivery of the intervention; adherence to the randomisation sequence by the PICUs; fidelity of uptake and acceptability of the intervention and determine whether any tweaks to the intervention are required.

For peer review only

PRISMA-P 2015 Checklist

This checklist has been adapted for use with protocol submissions to *Systematic Reviews* from Table 3 in Moher D et al: Preferred reporting items for systematic review and meta-analysis protocols (PRISMA-P) 2015 statement. *Systematic Reviews* 2015 **4**:1

Section/topic	#	Checklist item	Information reported		Line number(s)
			Yes	No	
**ADMINISTRATIVE INFORMATION**					
**Title**					
Identification	1a	Identify the report as a protocol of a systematic review	☐	☒	Extended systematic review
Update	1b	If the protocol is for an update of a previous systematic review, identify as such	☐	☒	NA
**Registration**	2	If registered, provide the name of the registry (e.g., PROSPERO) and registration number in the Abstract	☐	☒	NA
**Authors**					
Contact	3a	Provide name, institutional affiliation, and e-mail address of all protocol authors; provide physical mailing address of corresponding author	☒	☐	3-10
Contributions	3b	Describe contributions of protocol authors and identify the guarantor of the review	☒	☐	8, 320-324
**Amendments**	4	If the protocol represents an amendment of a previously completed or published protocol, identify as such and list changes; otherwise, state plan for documenting important protocol amendments	☐	☒	NA
**Support**					
Sources	5a	Indicate sources of financial or other support for the review	☒	☐	310-319
Sponsor	5b	Provide name for the review funder and/or sponsor	☒	☐	310-319
Role of sponsor/funder	5c	Describe roles of funder(s), sponsor(s), and/or institution(s), if any, in developing the protocol	☒	☐	310-319
**INTRODUCTION**					
**Rationale**	6	Describe the rationale for the review in the context of what is already known	☒	☐	69-138
**Objectives**	7	Provide an explicit statement of the question(s) the review will address with reference to	☒	☐	139-148

BioMed Central
The Open Access Publisher

For peer review only - http://bmjopen.bmj.com/site/about/guidelines.xhtml

Section/topic	#	Checklist item	Information reported Yes	Information reported No	Line number(s)
		participants, interventions, comparators, and outcomes (PICO)			
**METHODS**					
**Eligibility criteria**	8	Specify the study characteristics (e.g., PICO, study design, setting, time frame) and report characteristics (e.g., years considered, language, publication status) to be used as criteria for eligibility for the review	☒	☐	190-213
**Information sources**	9	Describe all intended information sources (e.g., electronic databases, contact with study authors, trial registers, or other grey literature sources) with planned dates of coverage	☒	☐	157-190
**Search strategy**	10	Present draft of search strategy to be used for at least one electronic database, including planned limits, such that it could be repeated	☒	☐	170, Table 1
STUDY RECORDS					
Data management	11a	Describe the mechanism(s) that will be used to manage records and data throughout the review	☒	☐	175-176, 218-219
Selection process	11b	State the process that will be used for selecting studies (e.g., two independent reviewers) through each phase of the review (i.e., screening, eligibility, and inclusion in meta-analysis)	☒	☐	163-169, 215-223
Data collection process	11c	Describe planned method of extracting data from reports (e.g., piloting forms, done independently, in duplicate), any processes for obtaining and confirming data from investigators	☒	☐	215-223
**Data items**	12	List and define all variables for which data will be sought (e.g., PICO items, funding sources), any pre-planned data assumptions and simplifications	☒	☐	224-266
**Outcomes and prioritization**	13	List and define all outcomes for which data will be sought, including prioritization of main and additional outcomes, with rationale	☒	☐	224-266
**Risk of bias in individual studies**	14	Describe anticipated methods for assessing risk of bias of individual studies, including whether this will be done at the outcome or study level, or both; state how this information will be used in data synthesis	☐	☒	NA
DATA					
**Synthesis**	15a	Describe criteria under which study data will be quantitatively synthesized	☐	☒	NA
	15b	If data are appropriate for quantitative synthesis, describe planned summary measures, methods of handling data, and methods of combining data from studies, including any planned exploration of consistency (e.g., I^2, Kendall's tau)	☐	☒	NA

BioMed Central
The Open Access Publisher

For peer review only

3

Section/topic	#	Checklist item	Information reported Yes	No	Line number(s)
	15c	Describe any proposed additional analyses (e.g., sensitivity or subgroup analyses, meta-regression)	☒	☐	267-270
	15d	If quantitative synthesis is not appropriate, describe the type of summary planned	☒	☐	267-270
**Meta-bias(es)**	16	Specify any planned assessment of meta-bias(es) (e.g., publication bias across studies, selective reporting within studies)	☐	☒	NA
**Confidence in cumulative evidence**	17	Describe how the strength of the body of evidence will be assessed (e.g., GRADE)	☐	☒	NA

For peer review only

BioMed Central The Open Access Publisher

BMJ Open

The use of feasibility studies for stepped-wedge cluster randomised trials: a protocol for a review of impact and scope.

Journal:	*BMJ Open*
Manuscript ID	bmjopen-2017-017290.R1
Article Type:	Protocol
Date Submitted by the Author:	29-Jun-2017
Complete List of Authors:	Kristunas, Caroline; University of Leicester, Department of Health Sciences<br>Hemming, Karla; University of Birmingham, Institute of Applied Health Research<br>Eborall, Helen; University of Leicester, Department of Health Sciences<br>Gray, Laura; University of Leicester, Department of Health Sciences
**Primary Subject Heading**:	Research methods
Secondary Subject Heading:	Public health
Keywords:	STATISTICS & RESEARCH METHODS, Stepped-wedge trial, Pilot and feasibility studies

SCHOLARONE™ Manuscripts

For peer review only

The use of feasibility studies for stepped-wedge cluster randomised trials: a protocol for a review of impact and scope

Caroline A Kristunas[1], Karla Hemming[2], Helen C Eborall[1], Laura J Gray[1]*

Author Affiliations:

[1]Department of Health Sciences, University of Leicester, Leicester, UK, LE1 7Rh.

[2]Institute of Applied Health Research, University of Birmingham, Birmingham, UK, B15 2TT.

**Corresponding author:* Laura J Gray; lg48@le.ac.uk, Department of Health Sciences, College of Medicine, Biological Sciences and Psychology, University of Leicester, Centre for Medicine, University Road, Leicester, LE1 7RH

For peer review only

ABSTRACT

Introduction: The stepped-wedge cluster randomised trial (SW-CRT) is a complex design, for which many decisions about key design parameters must be made during the planning. These include the number of steps and the duration of time needed to embed the intervention. Feasibility studies are likely to be useful for informing these decisions and increasing the likelihood of the main trial's success. However, the number of feasibility studies being conducted for SW-CRTs is currently unknown. This review aims to establish the number of feasibility studies being conducted for SW-CRTs and determine which feasibility issues are commonly investigated.

Methods and analysis: Fully published feasibility studies for SW-CRTs will be identified, according to pre-defined inclusion criteria, from searches conducted in Ovid MEDLINE, Scopus, EMBASE, and PsycINFO. In order to also identify and gain information on unpublished feasibility studies the following will be contacted: authors of published SW-CRTs (identified from the most recent systematic reviews); contacts for registered SW-CRTs (identified from clinical trials registries); lead statisticians of UK registered Clinical Trials Units; and researchers known to work in the area of SW-CRTs.

Data extraction will be conducted independently by two reviewers. For the fully published feasibility studies, data will be extracted on the study characteristics, the rationale for the study, the process for determining progression to a main trial, how the study informed the main trial and whether the main trial went ahead. The researchers involved in the unpublished feasibility studies will be contacted in order to elicit the same information.

A narrative synthesis will be conducted and provided alongside a descriptive analysis of the study characteristics.

Ethics and dissemination: This review does not require ethical approval, as no individual patient data will be used. The results of this review will be published in an open access pee-reviewed journal.

Strengths and limitations:

- This review will be the first to provide an insight into how feasibility studies are being used to inform SW-CRTs.
- This review will identify both published and unpublished feasibility studies for SW-CRTs.
- In order to ensure a robust review and minimise potential bias, the search strategy and inclusion and exclusion criteria have been pre-specified.
- Although steps have been taken to minimise potential sources of bias, selection bias may be introduced through the exclusion of non-English language studies.

For peer review only

INTRODUCTION

<u>The stepped-wedge cluster randomised trial</u>

The stepped-wedge cluster randomised trial (SW-CRT) is seeing an unprecedented increase in its use.[1-4] For this design, clusters are randomised to start the intervention at different time points. All of the clusters start the trial not receiving the intervention, and then switch to the intervention at their allocated start time, until by the end of the trial all of the clusters are receiving the intervention [Figure 1].[5]

There are several appeals of the SW-CRT. One of the main appeals being that it allows a staggered introduction of the intervention, allowing clusters to act as their own control and for all clusters to eventually receive the intervention.[2, 6] The introduction of the intervention is also randomised, making this design more desirable than a simple (non-randomised) before-and-after design, which are known to be confounded by temporal trends that cannot be adjusted for.[7] In addition, there are occasions when the SW-CRT is more efficient than a parallel CRT, requiring a smaller sample size and fewer clusters.[7, 8] The SW-CRT can thus allow the experimental assessment of the effectiveness of certain interventions that, due to practical, logistical or financial reasons, it may not be possible to assess using another design of trial.[5, 7, 9] An example of a study for which the SW-CRT was considered the best option is the SANDWICH trial.[10] Details of the reasons for this choice of design and the decision processes related to this choice are described in Figure 2.

<u>Trial decision process in the SW-CRT</u>

The staggered nature of the introduction of clusters to the intervention in a SW-CRT requires many decisions to be made during the design process, such as the number of start times and the length of time between start times.[7] For example there may be logistical constraints that mean it is only feasible to switch a certain number of clusters at once, or the intervention may take a while to embed in a cluster, in which case the length of time between start times might need to be sufficiently long to allow for this.[7] Alternatively, the trial might need to be completed within a fixed funding period, in which case the step length will need to be short enough to allow all clusters to switch within this period. These decisions are in addition to those that must be made for a standard CRT, and are unlikely to be able to be informed by previous studies. It is known that, although advantageous in some respects, the staggered introduction of the intervention may increase the complexity of the trial in both practical and statistical terms.[6, 7] Some of the issues associated with a staggered implementation are being investigated as part of a feasibility study for the SANDWICH trial, details of which are given in Figure 2.

<u>What feasibility and pilot studies may offer</u>

The terms pilot and feasibility study are often used interchangeably to describe a small scale study, that is conducted prior to the main trial, and which aims to guide the planning or design of the trial, or to confirm its feasibility.[11-14] When designing a trial, decisions may be informed by previous trials, systematic reviews, routine data etc., but there may be aspects of the trial that require additional information which might only be obtained through the use of a feasibility or pilot study.[13] Pilot or feasibility studies are becoming

For peer review only

the norm prior to a definitive individually randomised trial, and test trial factors such as recruitment, retention and acceptability of the intervention.[15, 16]

Cluster randomised trials (CRTs) are typically larger and more expensive, and due to their additional complexity, tend to need more assumptions and decisions to be made at the design stage than for an individually randomised trial.[13, 17] Each aspect of the design that is assessed at an individual-level, now needs to also be assessed at the cluster-level. These may include recruitment and retention rates or the acceptability of the intervention to both the individuals and the clusters. Specifically for CRTs, an estimate of the extent of variation between clusters (intracluster correlation coefficient (ICC)) is needed in order to calculate the required sample size. However, Eldridge et al. [17] have shown that feasibility studies to investigate ICCs for CRTs will often have insufficient accuracy, unless the feasibility study is almost as large as the main trial.

Feasibility studies may be of even greater potential benefit for SW-CRTs than they are for standard CRTs, as numerous additional decisions must be made. These include determining the number of clusters it is feasible to switch to the intervention at each step and the length of time between each step needed to embed the intervention, ensuring that the full effect of the intervention is realised and enough participants are recruited for each stage of the trial.[9]

Previous systematic reviews have investigated the reasons why a pilot or feasibility study might be undertaken for a randomised controlled trial (RCT),[11, 15, 18] but these reviews are unlikely to have captured many pilot or feasibility studies for SW-CRTs. In addition, no published systematic reviews of SW-CRTs have included feasibility studies, so it is not currently known to what extent feasibility studies are being conducted for SW-CRTs, nor what issues these studies are being designed to investigate.

What our review will achieve

We aim to identify what feasibility work is being conducted for SW-CRTs. This will be achieved through a systematic review of the published literature, which will identify published feasibility studies for SW-CRTs. However, as feasibility studies often go unpublished, we will extend our review to identify unpublished feasibility studies. This will be achieved by contacting the authors of published and registered SW-CRTs, lead statisticians of UK registered Clinical Trials Units (CTUs) and researchers who are known by the authors to work in the area of SW-CRTs.

OBJECTIVES

The overarching aims of this review are to determine the number of feasibility studies being conducted for SW-CRTs and how these studies are being used to inform the subsequent SW-CRTs. Specifically, our objectives are to:

1. Systematically identify published feasibility studies for SW-CRTs;
2. Identify unpublished feasibility work that has been conducted or is planned to take place prior to a SW-CRT;

For peer review only

3. Extract information on the design characteristics and rationale for these feasibility studies, as well as how these studies inform the main trial and how the decision is made as to whether to progress to the main trial.

METHODS

A two-pronged approach will be implemented to identify feasibility studies for SW-CRTs. Published feasibility studies will be identified through a systematic review of the literature, whereas unpublished feasibility studies will be identified by contacting researchers who may have been involved in, or have knowledge of, feasibility studies for SW-CRTs that have been conducted but which have not yet been published.

Search strategy

<u>Identification of published feasibility studies for SW-CRTs</u>

We will identify eligible feasibility studies for SW-CRTs, published in English, via electronic searches of the online published databases Ovid MEDLINE (1946-present), Scopus (1966-present), EMBASE (1947-present) and PsycINFO (1967-present). The initial search strategy will include a combination of MeSH terms and keywords specific to each bibliographic database. An example search strategy is outlined in Table 1 and is based on previously published search strategies.[1-3, 11, 15]

The titles and abstracts for the identified studies will be screened for eligibility by two reviewers independently in a random order, with full-text articles being obtained for potentially eligible studies and the same duplicate method of assessment used. Any studies which are not found to be eligible will be excluded and the reason for exclusion noted. Any differences of opinion will be resolved by a third reviewer if required. Authors will be contacted if any further information is required and we will aim to access any published or unpublished protocols for each of the identified feasibility studies.

Table 1: Example search strategy for Ovid MEDLINE

1. “pilot*”.mp
2. “feasibil*”.mp
3. 1 OR 2
4. “step* wedge*”.mp
5. “step*wedge*”.mp
6. “delay* intervention”.mp
7. “experimental* staged introduction”.mp
8. (“one* direction* crossover design” OR “one* direction* cross* over design”).mp
9. (“incremental* recruitment” OR “incremental* introduction” OR “incremental* implementation” OR “incremental* allocation”).mp
10. (“phased* recruitment” OR “phased* introduction” OR “phased* implementation” OR “phased* allocation”).mp
11. (“staggered* recruitment” OR “staggered* introduction” OR “staggered*

For peer review only

implementation" OR "staggered*allocation").mp

12. ("stepwise* recruitment" OR "stepwise* introduction" OR "stepwise* implementation" OR "stepwise*allocation").mp
13. ("step*wise* recruitment" OR "step*wise* introduction" OR "step*wise* implementation" OR "step*wise*allocation").mp
14. ("delayed* recruitment" OR "delayed* introduction" OR "delayed* implementation" OR "delayed*allocation").mp
15. or/4-14
16. 3 AND 15
17. limit 16 to English language

Reference lists of the identified studies will also be checked for potentially eligible studies. Any additional published feasibility studies for SW-CRTs which are known to the authors, but which are not identified during the database searches, will also be included and assessed for eligibility. Records will be managed using Refworks reference management software.

<u>Identification of unpublished feasibility studies for SW-CRTs</u>

Feasibility studies for SW-CRTs might not always be published. In an attempt to identify the majority of feasibility studies for SW-CRTs, our systematic review will be extended to allow inclusion of unpublished feasibility studies. This will be achieved in the following ways:

- Unpublished feasibility work for published SW-CRTs will be identified from the SW-CRTs reported in the most recent systematic reviews. The trial reports and references will be examined for evidence of feasibility work. The authors of these trials will then be contacted to identify whether any unreported feasibility work was conducted.
- Feasibility work that has been conducted for SW-CRTs that are yet to be published will be sought by contacting the lead statisticians of CTUs, researchers working in the area of stepped-wedge trials, as well as through searches of clinical trials registries.

Inclusion and exclusion criteria

For the systematic review of published feasibility studies, eligible studies will be full reports or protocols of feasibility studies for SW-CRTs published during the period covered by the searched databases (1946-present). For the unpublished feasibility studies no publications will be required in order to qualify for inclusion.

The definition of, and distinction between feasibility and pilot studies is not always clear, with the terms often being used interchangeably [11, 12] and conflicting definitions existing for the distinction between the terms.[14, 19] For the purpose of this review (including both published and unpublished studies) a feasibility study will be defined as a study, with clearly defined aims and objectives, which intends to ascertain the feasibility of a planned SW-CRT, through the assessment of issues other than solely the effectiveness and refinement of the

For peer review only

intervention, such as investigating recruitment issues. Since pilot studies will also often investigate issues affecting the feasibility of the main trial, they will be considered to be a type of feasibility study for the purpose of this review, provided they satisfy the criteria of a feasibility study.

The feasibility study itself does not need to be of a stepped-wedge design, or even randomised, as the design will depend on the objectives of the study. However, the study should, through focussed objectives, make it clear how the findings of the feasibility study will inform the main study, which must be intended to be of a SW-CRT design. A SW-CRT will be defined as a randomised trial, which randomises clusters and which has two or more steps (time-points at which clusters change treatment group). Studies where the planned definitive trial is individually randomised, has a bi-directional cross-over design or is non-randomised, will be excluded. Neither the published nor the unpublished feasibility studies will be limited to healthcare settings.

The researchers involved in the unpublished feasibility studies that we identify will be contacted to gain the information required to determine eligibility.

Data extraction

Data for eligible published feasibility studies will be extracted by two reviewers independently and in random order using a data extraction form that will have been tested on a small number of studies, before being refined and finalised. Any differences will be resolved though discussion with a third reviewer. Extracted data will be managed in Microsoft Excel 2013.

For the eligible unpublished feasibility studies, data will be obtained through semi-structured interviews with the researchers involved in the studies. This will be conducted by a single reviewer. The same data will be collected for the unpublished feasibility studies as is extracted for the published feasibility studies.

<u>Data extraction of trial characteristics</u>

Data will be extracted on aspects of the design of the feasibility study, including the recruitment, randomisation, blinding and overall design (parallel, stepped-wedge etc.), as well as how exposure to the intervention is experienced at an individual level. For example, in some studies subjects might receive both the control and intervention conditions, whereas in others they might only experience the one condition. Data will also be extracted on how each feasibility study defines itself (either as a pilot or feasibility study), the size of the feasibility study and how the chosen sample size is justified.

<u>Data extraction of study rationale</u>

Data will be extracted on the rationale for conducting a feasibility study prior to the main trial. This will be achieved by extracting the specific aims of each study and categorising them into process, resource, management and scientific motivations.[20] These categories will be defined as:

For peer review only

- Process- assesses the feasibility of the steps that need to take place during the main study. This will include the estimation of recruitment, consent and retention rates, testing of the process and acceptability of the randomisation procedure, testing of the exclusion criteria, identification of barriers to recruitment, adherence etc.. Some of these will be specific to the SW-CRT, for example the recruitment rates per measurement period, which will determine the length of time between steps, whereas others will be more generic.
- Resource- assesses the time and budget problems that may occur during the main trial. This may include the time taken for data collection e.g. time taken to mail or fill out forms/surveys and have them returned, or the time taken for data entry to be completed. The human resources available may also be a consideration, ensuring that the right people are available with the required expertise. Specific to SW-CRT, these aims might include the length of time required to rollout the intervention, or the time taken to acquire the necessary permissions from each of the clusters.
- Management- assesses the potential for human and data optimisation problems, such as the feasibility of international collaborations and across site coordination for multi-centre trials.
- Scientific motivations- assesses the scientific processes such as treatment safety and acceptability and estimation of the ICC (not recommended [17]), potential effectiveness and variance parameters.

In addition, the aims of each feasibility study will be sub-divided into aims that are specific to the SW-CRT and those which will be common with other trial designs.

Data extraction of progression to main trial

Data will be extracted on the process for deciding whether the study will progress to the main trial. This will include the type of analysis conducted, either hypothesis testing or descriptive, how much emphasis is put on the results of any hypothesis tests and the criteria used for determining success (main trial is considered feasible). Information on any hard or soft stopping rules that are in place for the feasibility studies will also be extracted.

Data extraction of main trial characteristics

Data will be extracted on the details of the main trials that follow each of the feasibility studies; whether it goes ahead, how the feasibility study has informed or made changes to the main trial and whether any of the participants who took part in the feasibility study also took part in the main trial.

Analysis of results

We will present a narrative synthesis of our findings, as well as a descriptive analysis of the study characteristics of each of the eligible feasibility studies that we identify for inclusion in the review.

Ethics and dissemination

For peer review only

As no individual patient data will be used, this review does not require ethical approval. We intend on presenting the findings of this review in an open-access peer-reviewed publication in an appropriate journal. We also intend on disseminating the findings through presentations at relevant conferences.

DISCUSSION

Although several reviews have been conducted in the area of stepped wedge trials,[1-4, 21, 22] none have investigated the use of feasibility studies for SW-CRTs. Due to their complexity, there are likely to be many issues that will affect the feasibility of SW-CRTs, some of which may be common across studies. However, it has yet to be reported how often feasibility work is being conducted prior to SW-CRTs, nor what feasibility issues these studies have been designed to inform. We aim to use our review to determine how many feasibility studies are conducted for SW-CRTs and what issues these studies have been designed to investigate. From the number of feasibility studies that we identify, we will be able to infer the rough order of magnitude of SW-CRTs which have some form of feasibility work conducted prior to the main trial. This will provide an estimate of how often feasibility studies are conducted prior to a SW-CRT.

By pre-specifying our search strategy, the inclusion and exclusion criteria and ensuring that two reviewers independently review all studies in a randomised order, we aim for our review to be robust, minimising potential sources of bias. Since the term “stepped wedge” has not been universally used to describe a trial of this design, we have included in our search strategy other terms used to describe this design, terms which have been used in previous reviews.[1-4, 22] However, there still remains the potential for selection bias in our review, as only trials using one of these terms will be included, which will exclude any SW-CRTs using alternative terminology. We may also introduce selection bias through the exclusion of non-English language studies, although there is evidence that this has a minimal effect.[23]

Our review would be limited if relying solely on published feasibility studies for SW-CRTs, as many will not have been published, particularly if proceeding to the main trial was regarded as infeasible. However, by extending our systematic review to identify unpublished feasibility studies for SW-CRTs, we will be able to widen the reach of our review. We will contact authors of published SW-CRTs and researchers known to be working in the area of SW-CRTs to determine if they have knowledge of additional studies that we can include in our review. It may be anticipated that there will be few feasibility studies for SW-CRTs. However, with the various methods we plan on implementing, we expect to be able to identify the majority of those feasibility studies for SW-CRTs that do exist. We have already identified a handful of these studies from a small scoping review and some studies that we already knew about. We are therefore optimistic that our full review will be able to identify further studies.

CONCLUSION

This review is the first in a series of related projects investigating the feasibility of SW-CRTs. It will determine the number of feasibility studies being conducted in order to inform SW-

For peer review only

CRTs and identify the feasibility issues that are being investigated and how these studies are informing the main SW-CRTs. This will provide an insight into how feasibility studies can benefit SW-CRTs. Future work will identify the feasibility issues being encountered in SW-CRTs and ultimately lead to the development of guidance on how feasibility studies for SW-CRTs can be conducted.

DECLARATIONS

Funding

This report is independent research supported by the National Institute for Health Research (NIHR Doctoral Research Fellowship, Miss Caroline Kristunas, DRF-2016-09-025. The authors would also like to acknowledge support from the NIHR Collaboration for Leadership in Applied Health Research and Care – East Midlands (NIHR CLAHRC – EM), NIHR CLAHRC – West Midlands, the Leicester Clinical Trials Unit and the NIHR Leicester-Loughborough Diet, Lifestyle and Physical Activity Biomedical Research Unit, which is a partnership between University Hospitals of Leicester NHS Trust, Loughborough University and the University of Leicester. The views expressed are those of the authors and not necessarily those of the NHS, the National Institute for Health Research or the Department of Health.

Contributors

CK conceptualised the review, the design of which was refined by incorporating comments from KH, HE and LG. CK drafted the manuscript and incorporated comments from KH, HE and LG for all subsequent drafts. All authors approved the final version and agree to be accountable for all aspects of the work.

Data sharing

No additional data are available.

Competing interests

We have read and understood BMJ policy on declaration of interests and declare that we have no competing interests.

Copyright

The Corresponding Author has the right to grant on behalf of all authors and does grant on behalf of all authors, a worldwide licence to the Publishers and its licensees in perpetuity, in all forms, formats and media (whether known now or created in the future), to i) publish, reproduce, distribute, display and store the Contribution, ii) translate the Contribution into other languages, create adaptations, reprints, include within collections and create summaries, extracts and/or, abstracts of the Contribution, iii) create any other derivative work(s) based on the Contribution, iv) to exploit all subsidiary rights in the Contribution, v) the inclusion of electronic links from the Contribution to third party material where-ever it may be located; and, vi) licence any third party to do any or all of the above.

For peer review only - http://bmjopen.bmj.com/site/about/guidelines.xhtml

REFERENCES

1 Brown CA, Lilford RJ. The stepped wedge trial design: a systematic review, *BMC medical research methodology* 2006;6:1.

2 Mdege ND, Man M, Taylor CA, et al. Systematic review of stepped wedge cluster randomized trials shows that design is particularly used to evaluate interventions during routine implementation, *J Clin Epidemiol* 2011;64:936-48.

3 Beard E, Lewis JJ, Copas A, et al. Stepped wedge randomised controlled trials: systematic review of studies published between 2010 and 2014, *Trials* 2015;16:1.

4 Grayling MJ, Wason JM, Mander AP. Stepped wedge cluster randomized controlled trial designs: a review of reporting quality and design features, *Trials* 2017;18:33.

5 Hussey MA, Hughes JP. Design and analysis of stepped wedge cluster randomized trials, *Contemporary clinical trials* 2007;28:182-91.

6 Prost A, Binik A, Abubakar I, et al. Logistic, ethical, and political dimensions of stepped wedge trials: critical review and case studies, *Trials* 2015;16:1.

7 Hemming K, Haines TP, Chilton PJ, et al. The stepped wedge cluster randomised trial: rationale, design, analysis, and reporting, *BMJ* 2015;350:h391 doi:10.1136/bmj.h391.

8 Baio G, Copas A, Ambler G, et al. Sample size calculation for a stepped wedge trial, *Trials* 2015;16:1.

9 Woertman W, de Hoop E, Moerbeek M, et al. Stepped wedge designs could reduce the required sample size in cluster randomized trials, *J Clin Epidemiol* 2013;66:752-8.

10 Anonymous . HTA - 15/104/01: Sedation and weaning in children: the SANDWICH trial. ;2017.

11 Arain M, Campbell MJ, Cooper CL, et al. What is a pilot or feasibility study? A review of current practice and editorial policy, *BMC Med Res Methodol* 2010;10:67.

12 Whitehead AL, Sully BGO, Campbell MJ. Pilot and feasibility studies: Is there a difference from each other and from a randomised controlled trial? *Contemporary Clinical Trials* 2014;38:130-3 doi:10.1016/j.cct.2014.04.001 [published Online First: May].

13 Eldridge S, Kerry S. A practical guide to cluster randomised trials in health services research: John Wiley & Sons 2012.

14 Eldridge SM, Chan CL, Campbell MJ, et al. CONSORT 2010 statement: extension to randomised pilot and feasibility trials, *Pilot and Feasibility Studies* 2016;2:64.

15 Lancaster GA, Dodd S, Williamson PR. Design and analysis of pilot studies: recommendations for good practice, *J Eval Clin Pract* 2004;10:307-12.

For peer review only

16 Anderson GL, Prentice RL. Individually randomized intervention trials for disease prevention and control, *Stat Methods Med Res* 1999;8:287-309.

17 Eldridge SM, Costelloe CE, Kahan BC, et al. How big should the pilot study for my cluster randomised trial be? *Statistical Methods in Medical Research* 2016;25:1039-56 doi:10.1177/0962280215588242 [published Online First: June 1,].

18 Shanyinde M, Pickering RM, Weatherall M. Questions asked and answered in pilot and feasibility randomized controlled trials, *BMC medical research methodology* 2011;11:117.

19 Eldridge SM, Lancaster GA, Campbell MJ, et al. Defining feasibility and pilot studies in preparation for randomised controlled trials: development of a conceptual framework, *PloS one* 2016;11:e0150205.

20 Thabane L, Ma J, Chu R, et al. A tutorial on pilot studies: the what, why and how, *BMC medical research methodology* 2010;10:1.

21 Barker D, McElduff P, D'Este C, et al. Stepped wedge cluster randomised trials: a review of the statistical methodology used and available, *BMC medical research methodology* 2016;16:1.

22 Martin J, Taljaard M, Girling A, et al. Systematic review finds major deficiencies in sample size methodology and reporting for stepped-wedge cluster randomised trials, *BMJ Open* 2016;6:010166.

23 Juni P, Holenstein F, Sterne J, et al. Direction and impact of language bias in meta-analyses of controlled trials: empirical study, *Int J Epidemiol* 2002;31:115-23.

Figure legends

Figure 1: Schematic of an example stepped-wedge cluster randomised trial (SW-CRTs), with four steps and one cluster switching from control (white) to the intervention (light blue) at each time point. A recruitment period (light green) has been included prior to the first measurement period, as well as a bedding-in period (dark blue) during which the intervention will be embedded within the cluster. A cross-sectional sample is taken during each measurement period, at the time point indicated by the orange line, from which the outcome measure is obtained. Not all SW-CRTs follow the design shown. For example, some SW-CRTs may not include a recruitment or bedding-in period and others may be of a cohort design.

Figure 2: A case study of the SANDWICH stepped-wedge cluster randomised trial.

For peer review only

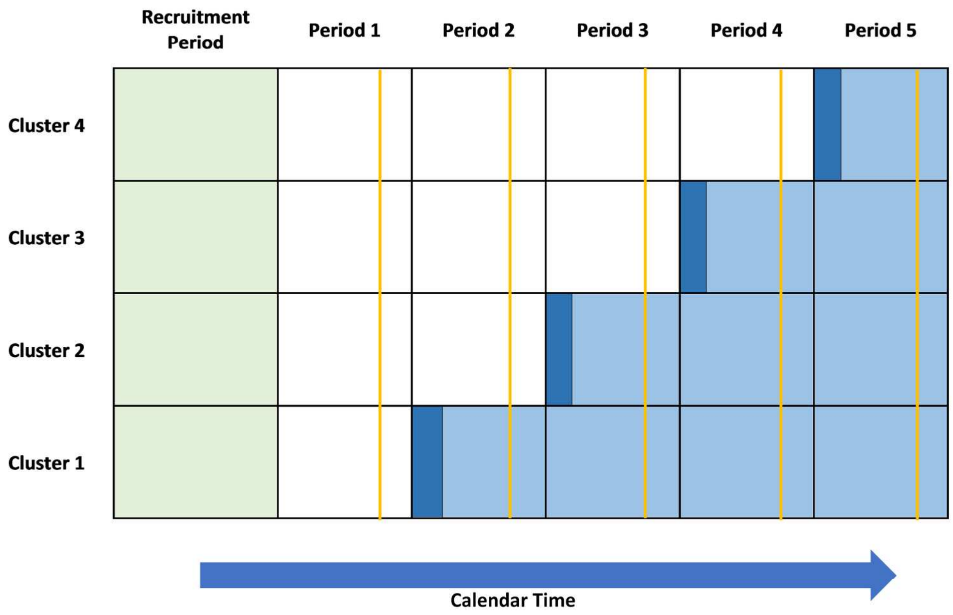

Schematic of an example stepped-wedge cluster randomised trial (SW-CRTs), with four steps and one cluster switching from control (white) to the intervention (light blue) at each time point. A recruitment period (light green) has been included prior to the first measurement period, as well as a bedding-in period (dark blue) during which the intervention will be embedded within the cluster. A cross-sectional sample is taken during each measurement period, at the time point indicated by the orange line, from which the outcome measure is obtained. Not all SW-CRTs follow the design shown. For example, some SW-CRTs may not include a recruitment or bedding-in period and others may be of a cohort design.

190x142mm (300 x 300 DPI)

only

Case study: the SANDWICH trial

The **S**edation **AND W**eaning **I**n **CH**ildren (SANDWICH) trial, will be a cluster-randomised stepped-wedge clinical and cost-effectiveness trial set in UK Paediatric Intensive Care Units (PICU). A protocol-based intervention, incorporating co-ordinated care with greater nursing involvement in managing sedation and weaning ventilation, will be assessed for its effectiveness at reducing the duration of invasive mechanical ventilation.

There are several reasons why a stepped-wedge cluster randomised design was chosen for the SANDWICH trial. Firstly, it was important that randomisation was undertaken at the cluster level, as the intervention will be delivered at the PICU (cluster) level and contamination would have occurred if individual-level randomisation had been used. Secondly, it would have been infeasible to conduct a conventional parallel cluster trial. There are a maximum of 20 PICUs in the UK, limiting the number of available clusters to such an extent that there would not have been a sufficient number to detect the important clinical effect at the desired 90% power.

It is anticipated that, due to the staggered rollout of the intervention, there will be particular issues that will need to be overcome. It is desired to conceal the allocation sequence, as advance knowledge of the allocation sequence can result in clusters withdrawing if they are allocated to switch to the intervention late in the trial. A feasibility study, taking the form of an internal pilot, will be used to determine how close the crossover date can be revealed to each cluster, to allow them sufficient time to prepare. This study will also aim to confirm the feasibility of data collection procedures and delivery of the intervention; adherence to the randomisation sequence by the PICUs; fidelity of uptake and acceptability of the intervention and determine whether any tweaks to the intervention are required.

A case study of the SANDWICH stepped-wedge cluster randomised trial.

190x142mm (300 x 300 DPI)

PRISMA-P 2015 Checklist

This checklist has been adapted for use with protocol submissions to *Systematic Reviews* from Table 3 in Moher D et al: Preferred reporting items for systematic review and meta-analysis protocols (PRISMA-P) 2015 statement. *Systematic Reviews* 2015 **4**:1

Section/topic	#	Checklist item	Information reported Yes	Information reported No	Line number(s)
**ADMINISTRATIVE INFORMATION**					
**Title**					
Identification	1a	Identify the report as a protocol of a systematic review	☐	☒	Extended systematic review
Update	1b	If the protocol is for an update of a previous systematic review, identify as such	☐	☒	NA
**Registration**	2	If registered, provide the name of the registry (e.g., PROSPERO) and registration number in the Abstract	☐	☒	NA
**Authors**					
Contact	3a	Provide name, institutional affiliation, and e-mail address of all protocol authors; provide physical mailing address of corresponding author	☒	☐	3-10
Contributions	3b	Describe contributions of protocol authors and identify the guarantor of the review	☒	☐	8, 344-348
**Amendments**	4	If the protocol represents an amendment of a previously completed or published protocol, identify as such and list changes; otherwise, state plan for documenting important protocol amendments	☐	☒	NA
**Support**					
Sources	5a	Indicate sources of financial or other support for the review	☒	☐	334-343
Sponsor	5b	Provide name for the review funder and/or sponsor	☒	☐	334-343
Role of sponsor/funder	5c	Describe roles of funder(s), sponsor(s), and/or institution(s), if any, in developing the protocol	☒	☐	334-343
**INTRODUCTION**					
**Rationale**	6	Describe the rationale for the review in the context of what is already known	☒	☐	69-140
**Objectives**	7	Provide an explicit statement of the question(s) the review will address with reference to	☒	☐	142-150

For peer review only

BioMed Central The Open Access Publisher

2

Section/topic	#	Checklist item	Information reported Yes	Information reported No	Line number(s)
		participants, interventions, comparators, and outcomes (PICO)			
**METHODS**					
**Eligibility criteria**	8	Specify the study characteristics (e.g., PICO, study design, setting, time frame) and report characteristics (e.g., years considered, language, publication status) to be used as criteria for eligibility for the review	☒	☐	194-218
**Information sources**	9	Describe all intended information sources (e.g., electronic databases, contact with study authors, trial registers, or other grey literature sources) with planned dates of coverage	☒	☐	157-192
**Search strategy**	10	Present draft of search strategy to be used for at least one electronic database, including planned limits, such that it could be repeated	☒	☐	172, Table 1
STUDY RECORDS					
Data management	11a	Describe the mechanism(s) that will be used to manage records and data throughout the review	☒	☐	177-178, 223-224
Selection process	11b	State the process that will be used for selecting studies (e.g., two independent reviewers) through each phase of the review (i.e., screening, eligibility, and inclusion in meta-analysis)	☒	☐	165-171, 220-228
Data collection process	11c	Describe planned method of extracting data from reports (e.g., piloting forms, done independently, in duplicate), any processes for obtaining and confirming data from investigators	☒	☐	220-228
**Data items**	12	List and define all variables for which data will be sought (e.g., PICO items, funding sources), any pre-planned data assumptions and simplifications	☒	☐	229-274
**Outcomes and prioritization**	13	List and define all outcomes for which data will be sought, including prioritization of main and additional outcomes, with rationale	☒	☐	229-274
**Risk of bias in individual studies**	14	Describe anticipated methods for assessing risk of bias of individual studies, including whether this will be done at the outcome or study level, or both; state how this information will be used in data synthesis	☐	☒	NA
DATA					
**Synthesis**	15a	Describe criteria under which study data will be quantitatively synthesized	☐	☒	NA
	15b	If data are appropriate for quantitative synthesis, describe planned summary measures, methods of handling data, and methods of combining data from studies, including any planned exploration of consistency (e.g., I^2, Kendall's tau)	☐	☒	NA

BioMed Central The Open Access Publisher

For peer review only

Section/topic	#	Checklist item	Information reported		Line number(s)
			Yes	No	
	15c	Describe any proposed additional analyses (e.g., sensitivity or subgroup analyses, meta-regression)	☒	☐	275-278
	15d	If quantitative synthesis is not appropriate, describe the type of summary planned	☒	☐	275-278
**Meta-bias(es)**	16	Specify any planned assessment of meta-bias(es) (e.g., publication bias across studies, selective reporting within studies)	☐	☒	NA
**Confidence in cumulative evidence**	17	Describe how the strength of the body of evidence will be assessed (e.g., GRADE)	☐	☒	NA

For peer review only

BioMed Central The Open Access Publisher
